# Supplementary material for: New insights into the Devonian sea spiders of the Hunsrück Slate (Arthropoda: Pycnogonida)
Source: PeerJ. 2024 Oct 14;12:e17766. doi: 10.7717/peerj.17766 (PMC11485130; doi:10.7717/peerj.17766)
Supplement: Supplemental Information 1 — For each plate the number of specimens is indicated, as well as the hosting collection (IGPB = Institut für Geowissenschaften (Section Palaeontology), Universität Bonn; MB = Museum für Naturkunde, Berlin; NHMMZ = Naturhistorisches Museum Mainz; BSPG = Bayerische Staatssammlung für Paläontologie und Geologie, Munich), associated species found with the pycnogonids, locality, stratigraphy, number of specimens, taxonomic status, study method used, previous illustrations in the literature, and citation in Bergström, Stürmer & Winter (1980) when applicable. [file peerj-12-17766-s001.docx]

**Table S1.** List of studied fossil slabs. For each plate the number of specimens is indicated, as well as the hosting collection (IGPB = Institut für Geowissenschaften (Section Palaeontology), Universität Bonn; MB = Museum für Naturkunde, Berlin; NHMMZ = Naturhistorisches Museum Mainz; BSPG = Bayerische Staatssammlung für Paläontologie und Geologie, Munich), associated species found with the pycnogonids, locality, stratigraphy, number of specimens, taxonomic status, study method used, previous illustrations in the literature, and citation in Bergström, Stürmer & Winter (1980) when applicable.

| **collection number** | **Hosting collection** | **species** | **association** | **locality** | **stratigraphy** | **# specimens** | **status** | **CT** | **RTI** | **Previously illustrated** | **Id number in** Bergström, Stürmer & Winter, 1980 |
| --- | --- | --- | --- | --- | --- | --- | --- | --- | --- | --- | --- |
| IGPB-AR339 | IGPB | *Palaeoisopus problematicus* |  | Bundenbach | middle Kaub Formation | 1 |  |  | X |  |  |
| IGPB-AR-340 | IGPB | *Palaeoisopus problematicus* |  | Bundenbach | middle Kaub Formation | 1 |  |  | X |  |  |
| IGPB-HS206 | IGPB | *Palaeoisopus problematicus* |  | Bundenbach, Eschenbach quarry | middle Kaub Formation | 1 |  | X | X | Kühl et al. 2012a, b |  |
| IGPB-HS207 | IGPB | *Palaeoisopus problematicus* |  | Bundenbach, Eschenbach quarry | middle Kaub Formation | 1 |  | X | X |  |  |
| IGPB-HS437 | IGPB | Pycnogonida gen. sp. |  | Bundenbach, Eschenbach quarry | middle Kaub Formation | 1 |  | X | X | Bartels, Briggs & Brassel, 1998 |  |
| IGPB-HS456 | IGPB | *Palaeoisopus problematicus* |  | Bundenbach, Eschenbach quarry | middle Kaub Formation | 1 |  |  | X | Bartels, Briggs & Brassel, 1998 |  |
| IGPB-HS457 | IGPB | *Palaeoisopus problematicus* |  | Bundenbach, Eschenbach quarry | middle Kaub Formation | 1 |  |  | X |  |  |
| IGPB-HS582 | IGPB | *Palaeoisopus problematicus* |  | Bundenbach, Eschenbach-Bocksberg quarry | middle Kaub Formation | 2 |  |  | X | Kühl et al., 2012a, b ; Rust et al., 2016 |  |
| IGPB-HS636 | IGPB | *Palaeoisopus problematicus* |  | Bundenbach, Eschenbach-Bocksberg quarry | middle Kaub Formation | 1 |  | X | X |  |  |
| IGPB-HS660 | IGPB | *Palaeoisopus problematicus* |  | Bundenbach, Eschenbach-Bocksberg quarry | middle Kaub Formation | 1 |  |  | X |  |  |
| IGPB-HS694 | IGPB | *Palaeoisopus problematicus* |  | Bundenbach, Eschenbach-Bocksberg quarry | middle Kaub Formation | 1 |  |  | X |  |  |
| IGPB-HS942 | IGPB | *Palaeoisopus problematicus* + Pycnogonida indet. | *Hapalocrinus frechi* Jäkel, 1895 | Bundenbach, Obereschenbach quarry | Wingertshell Member | 2 |  | X | X | Kühl, 2011 [‘HS145’] |  |
| IGPB-HS1039 | IGPB | *Palaeoisopus problematicus* |  | Bundenbach, Obereschenbach quarry | Wingertshell Member | 3 |  |  | X |  |  |
| IGPB-M-142 | IGPB | *Palaeoisopus problematicus* |  | Bundenbach, Schmiedenberg quarry | middle Kaub Formation | 1 |  |  | X | Kühl, 2011 |  |
| MB-A-288 | MB | *Palaeoisopus problematicus* |  | Germünden | middle Kaub Formation | 1 |  |  | X |  |  |
| MB-A-313 | MB | *Palaeoisopus problematicus* |  | Bundenbach? | middle Kaub Formation? | 1 |  |  | X |  |  |
| MB-A-3969 | MB | *Palaeoisopus problematicus* |  | Germünden | middle Kaub Formation | 1 |  |  | X |  |  |
| MB-A-45 | MB | *Palaeopantopus maucheri* |  | Germünden | middle Kaub Formation | 1 |  | X | X | Bergström, Stürmer & Winter, 1980 |  |
| MB-A-46 | MB | *Palaeoisopus problematicus* |  | Germünden | middle Kaub Formation | 1 |  |  | X | Bergström, Stürmer & Winter, 1980 | WS4744 |
| MB-A-47 | MB | *Palaeoisopus problematicus* |  | Germünden | middle Kaub Formation | 1 |  |  | X |  |  |
| NHMMZ PWL 1986/3 | NHMMZ | *Palaeoisopus problematicus* | *Hapalocrinus* sp. | Bundenbach | middle Kaub Formation | 1 |  |  | X | Kühl, 2011 |  |
| NHMMZ PWL 1992/178-LS | NHMMZ | *Palaeoisopus problematicus* |  | Bundenbach, Eschenbach quarry | middle Kaub Formation | 1 |  |  | X |  |  |
| NHMMZ PWL 1994/54-LS | NHMMZ | *Palaeoisopus problematicus* |  | Bundenbach | middle Kaub Formation | 2 |  |  | X | Kühl, 2011 |  |
| NHMMZ PWL 1994/55-LS | NHMMZ | *Palaeoisopus problematicus* |  | Bundenbach | middle Kaub Formation | 1 |  |  | X |  |  |
| NHMMZ PWL 1994/56-LS | NHMMZ | *Palaeoisopus problematicus* |  | Bundenbach | middle Kaub Formation | 1 |  |  | X | Kühl, 2011 |  |
| NHMMZ PWL 1994/133-LS | NHMMZ | *Palaeoisopus problematicus* |  | Bundenbach | middle Kaub Formation | 1 |  |  | X |  |  |
| NHMMZ PWL 1995/17-LS | NHMMZ | *Palaeoisopus problematicus* |  | Bundenbach? | middle Kaub Formation? | 1 |  |  | X |  |  |
| NHMMZ PWL 1995/35-LS | NHMMZ | *Palaeoisopus problematicus* |  | Bundenbach | middle Kaub Formation | 4 |  |  | [part] |  |  |
| NHMMZ PWL 1996/18-LS | NHMMZ | *Palaeoisopus problematicus* |  | Bundenbach | middle Kaub Formation | 1 |  | X | X | Lehmann 1959,  Bergström, Stürmer & Winter, 1980 | WS4779 |
| NHMMZ PWL 1997/44-45-LS | NHMMZ | *Palaeoisopus problematicus* |  | Bundenbach | middle Kaub Formation | 3 |  |  | X |  |  |
| NHMMZ PWL 1998/122-LS | NHMMZ | *Palaeoisopus problematicus* |  | Bundenbach | middle Kaub Formation | 2 |  |  | [part] |  |  |
| NHMMZ PWL 1998/155-LS | NHMMZ | *Palaeoisopus problematicus* |  | Bundenbach | Wingertshell Member? | 1 |  |  | X |  |  |
| NHMMZ PWL 2000/46 | NHMMZ | *Palaeoisopus problematicus* | *Parisangulocrinus minax* (Schmidt, 1934)  *+ Aulopora* sp. | Bundenbach | middle Kaub Formation | 1 |  |  | X | Kühl, 2011 |  |
| NHMMZ PWL 2003/272-LS | NHMMZ | *Palaeoisopus problematicus* |  | Bundenbach, Eschenbach-Bocksberg quarry | middle Kaub Formation | 3 |  |  | X | Kühl, 2011 |  |
| NHMMZ PWL 2004/5024-LS | NHMMZ | *Flagellopantopus blocki* |  | Obereschenbach quarry?  Eschenbach-Bocksberg quarry? | middle Kaub Formation  Wingertshell Member? | 1 | HOLOTYPE | X | X | Poschmmann & Dunlop, 2006 |  |
| NHMMZ PWL 2007/29-LS | NHMMZ | *Pentapantopus*? *vogteli*? | *Bundenbachia beneckei* Stürtz, 1886 | Bundenbach | middle Kaub Formation | 1 |  | X | X | Kühl, 2011 |  |
| NHMMZ PWL 2008/141-LS | NHMMZ | *Palaeoisopus problematicus* |  | Bundenbach, Eschenbach-Bocksberg quarry | middle Kaub Formation | 1 |  | X | X | Kühl, 2011 |  |
| NHMMZ PWL 2010/5-LS | NHMMZ | *Pentapantopus vogteli* + Pycnogonida indet. | *Parisangulocrinus zeaeformis* (Follmann, 1887) | Obereschenbach quarry?  Eschenbach-Bocksberg quarry? | middle Kaub Formation  Wingertshell Member? | 5 | HOLOTYPE & PARATYPE [part] | X | [part] | Kühl, 2011; Kühl, Poschmann & Rust, 2013 |  |
| NHMMZ PWL 2013/8 | NHMMZ | *Palaeoisopus problematicus* | *Retifungus rugens* Rietschel, 1970 | Bundenbach | middle Kaub Formation | 1 |  |  | X |  |  |
| SNSB-BSPG 1928 VII 11 | BSPG | *Palaeoisopus problematicus* | *Hapalocrinus frechi* Jäkel, 1895 | Bundenbach | middle Kaub Formation | 1 | HOLOTYPE | X | X | Broili 1928; Helfer 1932; Bergström, Stürmer & Winter, 1980 | WS2807 |
| SNSB-BSPG 1929 V 3 | BSPG | *Palaeopantopus maucheri* |  | Bundenbach | middle Kaub Formation | 1 | HOLOTYPE | X | X | Broili 1929,  Bergström, Stürmer & Winter, 1980 | WS2812 |
| SNSB-BSPG 1930 I 501 | BSPG | *Palaeopantopus maucheri* |  | Bundenbach | middle Kaub Formation | 1 |  | X | X | Broili 1930,  Bergström, Stürmer & Winter, 1980 | WS2810 |
| SNSB-BSPG 1932 I 63 | BSPG | *Palaeoisopus problematicus* |  | Bundenbach | middle Kaub Formation | 1 |  | X | X | Lehmann 1959,  Bergström, Stürmer & Winter, 1980 | WS2808 |
| SNSB-BSPG 1932 I 67 | BSPG | *Palaeoisopus problematicus* |  | Bundenbach | middle Kaub Formation | 1 |  | X | X | Lehmann 1959,  Bergström, Stürmer & Winter, 1980 | WS2813  [‘WS2313’ in fig. 8] |
| SNSB-BSPG 1967 I 306 | BSPG | *Palaeoisopus problematicus* |  | Bundenbach | middle Kaub Formation | 1 |  | X | X | Bergström, Stürmer & Winter, 1980 | WS2814 |
| SNSB-BSPG 2021 IV 1 | BSPG | *Palaeoisopus problematicus* |  | ? | ? | 1 |  |  | X | Bergström, Stürmer & Winter, 1980 | WS592 |
