# Supplementary material for: New insights into the Devonian sea spiders of the Hunsrück Slate (Arthropoda: Pycnogonida)
Source: PeerJ. 2024 Oct 14;12:e17766. doi: 10.7717/peerj.17766 (PMC11485130; doi:10.7717/peerj.17766)
Supplement: Supplemental Information 2 — All data available in the on-line material. [file peerj-12-17766-s002.docx]

**Table S2**. X-ray tomography parameters used in this study and studied material. All data available in the on-line material.

| **species** | **specimen** | **figures** | **CT-scanner** | **Current (µA)** | **Voltage (kV)** | **Exposure time (ms)** | **Filter type** | **Final resolution (µm)** |
| --- | --- | --- | --- | --- | --- | --- | --- | --- |
| ***Palaeoisopus problematicus*** | SNSB-BSPG 1928 VII 11 | Fig. 1D-E | Nikon XTH 225ST | 184 | 215 | 1415 | 2mm Cu | 62.6 |
|  | SNSB-BSPG 1932 I 63 | - | Nikon XTH 225ST | 271 | 190 | 2000 | none | 115.8 |
|  | SNSB-BSPG 1932 I 67 | - | Nikon XTH 225ST | 271 | 190 | 2000 | none | 115.8 |
|  | SNSB-BSPG 1967 I 306 | - | Phoenix\|x-ray v\|tome\|xs | 300 | 30 | 1000 | none | 73.1 |
|  | IGPB-HS206 | - | Nikon XTH 225ST | 300 | 182 | 2000 | 1mm Tn | 126.1 |
|  | IGPB-HS207 | - | Nikon XTH 225ST | 300 | 182 | 2000 | 1mm Tn | 126.1 |
|  | IGPB-HS636 | - | Nikon XTH 225ST | 300 | 182 | 2000 | 1mm Tn | 126.1 |
|  | IGPB-HS942 | - | Nikon XTH 225ST | 184 | 215 | 1415 | 2mm Cu | 62.6 |
|  | NHMMZ PWL 1996/18-LS | - | Phoenix\|x-ray v\|tome\|xs | 300 | 30 | 1000 | none | 114.1 |
|  | NHMMZ PWL 2008/141-LS | - | Phoenix\|x-ray v\|tome\|xs | 300 | 40 | 1000 | none | 114.1 |
| ***Palaeopantopus maucheri*** | SNSB-BSPG 1929 V 3 | Fig. 20G | Phoenix\|x-ray v\|tome\|xs | 200 | 50 | 1000 | none | 69.6 |
|  | SNSB-BSPG 1930 I 501 | - | Nikon XTH 225ST | 206 | 213 | 2000 | 1mm Tn | 49.1 |
|  | MB-A-45 | Fig. 22E | Yxlon FF85 Modular | 70 | 130 | 1000 | 0.2 mm Cu | 10.0 |
| **Pycnogonida gen. sp.** | IGPB-HS437 | Figs 32C, 33D-F | Nikon XTH 225ST | 184 | 215 | 1415 | 2mm Cu | 62.6 |
| ***Flagellopantopus blocki*** | NHMMZ PWL 2004/5024-LS | Figs 25D-E, 26E, F | Phoenix\|x-ray v\|tome\|xs | 300 | 100 | 1000 | none | 41.1 |
| ***Pentapantopus vogteli*** | NHMMZ PWL 2010/5-LS, specimens 1, 2 | Fig. 28C | Phoenix\|x-ray v\|tome\|xs | 300 | 40 | 1000 | none | 32.3 |
|  | NHMMZ PWL 2010/5-LS, specimen 3 | Fig. 29A, C-E |  |  |  |  |  |  |
| ***Pentapantopus? vogteli?*** | NHMMZ PWL 2007/29-LS | - | Phoenix\|x-ray v\|tome\|xs | 300 | 50 | 1000 | none | 32.2 |
| **Pycnogonida indet** | NHMMZ PWL 2010/5-LS, specimens 1, 2 | - | Phoenix\|x-ray v\|tome\|xs | 300 | 40 | 1000 | none | 32.3 |
