# Supplementary material for: New insights into the Devonian sea spiders of the Hunsrück Slate (Arthropoda: Pycnogonida)
Source: PeerJ. 2024 Oct 14;12:e17766. doi: 10.7717/peerj.17766 (PMC11485130; doi:10.7717/peerj.17766)
Supplement: Supplemental Information 3 — Photos were taken with a Nikon D850 mounted with NIKKOR 40 (“40”), 60 (“60”) and 105 (“105”) mm lenses or a Canon EOS 700D digital camera, with Canon 50 (“50”) mm and Canon EF 100 (“100”) mm macro lenses. When in-text figures provide specular enhancement views, parameters to produce these images on RTIviewer (Cultural Heritage Imaging, San Francisco) are described. The RTI number column indicates the file number of the on-line material. [file peerj-12-17766-s003.docx]

**Table S3.** List of RTI files. Photos were taken with a Nikon D850 mounted with NIKKOR 40 (“40”), 60 (“60”) and 105 (“105”) mm lenses or a Canon EOS 700D digital camera, with Canon 50 (“50”) mm and Canon EF 100 (“100”) mm macro lenses. When in-text figures provide specular enhancement views, parameters to produce these images on RTIviewer (Cultural Heritage Imaging, San Francisco) are described. The RTI number column indicates the file number of the on-line material.

| **species** | **specimen(s)** | **RTI n°** | **Picture focus** | **camera lens** | **specular enhancement parameters** | | | | | |
| --- | --- | --- | --- | --- | --- | --- | --- | --- | --- | --- |
|  |  |  |  |  | **figure** | **diffuse color** | **specularity** | **highlight size** | **light orientation (x)** | **light orientation (y)** |
| *Palaeoisopus problematicus* | SNSB-BSPG 2021 IV 1 | 1 | legs and body fragments | 60 | - | - | - | - | - | - |
|  |  | 2 | legs and body fragments | 60 | - | - | - | - | - | - |
|  | SNSB-BSPG 1928 VII 11 | - | whole specimen | 50 | fig. 1 A-C | 0 | 43 | 26 | 0.29 | 0.44 |
|  | SNSB-BSPG 1932 I 63 | - | body | 50 | fig. 12 G, N, U | 82 | 60 | 150 | 0.53 | 0.59 |
|  |  |  |  |  | fig. 13 B, G, L | 52 | 60 | 17 | 0.36 | -0.53 |
|  | SNSB-BSPG 1932 I 67 | - | cephalon, proboscis, cephalic appendages and trunk, ventral | 50 | fig. 3 C, G, K | 30 | 42 | 95 | -0.51 | -0.64 |
|  |  |  |  |  | fig. 11 C, F, I | 16 | 51 | 135 | 0.32 | -0.03 |
|  | SNSB-BSPG 1967 I 306 | 1 | cephalon and trunk, ventral | 60 | - | - | - | - | - | - |
|  |  | 2 | WL1-4 | 60 | - | - | - | - | - | - |
|  | NHMMZ PWL 1986/3 | - | whole specimen, dorsal? | 60 | fig. 18 E-G | 20 | 70 | 45 | 0.02 | 0.17 |
|  | NHMMZ PWL 1992/178-LS | 1 | body | 60 | - | - | - | - | - | - |
|  |  | 2 | abdomen | 60 | - | - | - | - | - | - |
|  | NHMMZ PWL 1994/54-LS | 1 | whole specimen, dorsal, specimen 1 (+ parts specimen 2) | 60 | fig. 18 I-K | 0 | 67 | 23 | -0.17 | 0.28 |
|  |  | 2 | body, specimen 1 | 105 | - | - | - | - | - | - |
|  | NHMMZ PWL 1994/55-LS | 1 | body, dorsal | 60 | - | - | - | - | - | - |
|  |  | 2 | right WL | 60 | - | - | - | - | - | - |
|  |  | 3 | right oviger, distal podomeres | 105 | - | - | - | - | - | - |
|  |  | 4 | left oviger, distal podomeres | 105 | - | - | - | - | - | - |
|  |  | 5 | chelifores | 105 | - | - | - | - | - | - |
|  | NHMMZ PWL 1994/56-LS | 1 | abdomen, lateral | 60 | fig. 9 A-B | - | - | - | - | - |
|  |  | 2 | trunk and cephalon, lateral | 60 | fig. 13 C, H, M | 0 | 80 | 34 | -0.72 | 0.60 |
|  | NHMMZ PWL 1994/133-LS | 1 | body, dorsal | 60 | fig. 2 A-C | 20 | 48 | 78 | 0.02 | 0.39 |
|  |  |  |  |  | fig. 13 D, I, N | 0 | 50 | 82 | 0.37 | -0.42 |
|  |  |  |  |  | fig. 13 E, J, O | 0 | 50 | 102 | 0.40 | 0.34 |
|  |  | 2 | cephalon, dorsal | 60 | fig. 5 A, D, G | 40 | 70 | 75 | -0.79 | 0.08 |
|  | NHMMZ PWL 1995/17-LS | 1 | body | 60 | - | - | - | - | - | - |
|  |  | 2 | abdomen | 60 | - | - | - | - | - | - |
|  |  | 3 | right WL4 | 60 | - | - | - | - | - | - |
|  |  | 4 | right WLs2-4 | 60 | - | - | - | - | - | - |
|  | NHMMZ PWL 1995/35-LS | 1 | specimen 1, body, lateral | 60 | fig. 4B, D | - | - | - | - | - |
|  |  |  |  |  | fig. 12 D, K, R | 4 | 93 | 39 | -0.01 | 0.10 |
|  |  | 2 | specimen 1, abdomen and WLs | 60 | - | - | - | - | - | - |
|  |  | 3 | specimen 2, body | 60 | fig. 12 E, L, S | 40 | 70 | 75 | 0.56 | 0.04 |
|  |  | 4 | specimen 2, abdomen, lateral | 60 | fig. 9 D, E | - | - | - | - | - |
|  |  | 5 | specimen 3 body | 60 | fig. 13 A, F, K | 40 | 60 | 75 | 0.06 | 0.2 |
|  |  | 6 | specimen 3, abdomen, lateral | 60 | fig. 9 G, H | - | - | - | - | - |
|  | NHMMZ PWL 1996/18-LS | 1 | chelifores | 105 | fig. 11 A, D, G | 29 | 56 | 35 | -0.28 | -0.39 |
|  |  | 2 | right palp | 105 | fig. 12 A, H, O | 14 | 93 | 36 | -0.38 | 0.21 |
|  |  | 3 | left palp | 105 | fig. 12 B, I, P | 36 | 94 | 108 | 0.42 | -0.37 |
|  |  | 4 | trunk and cephalon, dorsal | 60 | - | - | - | - | - | - |
|  |  | 5 | abdomen, dorsal | 60 | - | - | - | - | - | - |
|  |  | 6 | right WL1 | 60 | - | - | - | - | - | - |
|  |  | 7 | right WL2, 3 | 60 | - | - | - | - | - | - |
|  | NHMMZ PWL 1997/44-45-LS | 1 | cephalon, ventral specimen 1 | 60 | fig. 3 D, H, L | 10 | 85 | 59 | 0.16 | 0.48 |
|  |  |  |  |  | fig. 12 F, M, T | 69 | 60 | 47 | 0.71 | 0.16 |
|  |  | 2 | abdomen specimen 2 | 60 | - | - | - | - | - | - |
|  |  | 3 | trunk and cephalon dorsal, specimen 2 (+ WL1 specimens 1 and 3) | 60 | - | - | - | - | - | - |
|  | NHMMZ PWL 1998/122-LS | 1 | abdomen, dorsal, specimen 1 | 60 | fig. 8 E-G | 0 | 55 | 25 | 0.5 | -0.13 |
|  |  | 2 | body dorsal, specimen 1 | 60 | - | - | - | - | - | - |
|  | NHMMZ PWL 1998/155-LS | - | body, lateral | 60 | - | - | - | - | - | - |
|  | NHMMZ PWL 2000/46 | - | whole specimen, ventral | 60 | fig. 18 A-C | 20 | 61 | 65 | 0.27 | 0.22 |
|  | NHMMZ PWL 2003/272-LS | 1 | whole specimen, ventral, specimen 1 | 60 | fig. 17 B, D, F | 0 | 75 | 35 | -0.38 | 0.52 |
|  |  | 2 | terminal WL1, specimen 2 | 60 | - | - | - | - | - | - |
|  |  | 3 | fraction of WL1, 2 and palp, specimen 3 | 60 | - | - | - | - | - | - |
|  | NHMMZ PWL 2008/141-LS | 1 | cephalon and first trunk segment, dorsal | 60 | fig. 5 B, E, H | 16 | 70 | 106 | 0.08 | 0.83 |
|  |  | 2 | head and chelifores | 105 | fig. 11 B, E, H | 27 | 73 | 54 | 0.63 | 0.07 |
|  |  | 3 | left palp | 105 | fig. 12 C, J, Q | 10 | 60 | 106 | 0.31 | -0.33 |
|  |  | 4 | right palp | 105 | - | - | - | - | - | - |
|  |  | 5 | left WL1 | 60 | fig. 15 A-C | 0 | 82 | 16 | -0.52 | -0.22 |
|  |  | 6 | right WL2-4 | 60 | - | - | - | - | - | - |
|  |  | 7 | left WL2-4 | 60 | - | - | - | - | - | - |
|  | NHMMZ PWL 2013/8-LS | 1 | body, ventral | 60 | - | - | - | - | - | - |
|  |  | 2 | left WL2-4 | 60 | - | - | - | - | - | - |
|  |  | 3 | chelifores, ventral | 105 | - | - | - | - | - | - |
|  | IGPB-AR-339 | 1 | Cephalon and cephalic appendages | 60 | - | - | - | - | - | - |
|  |  | 2 | Left WL1, 2 | 60 | - | - | - | - | - | - |
|  |  | 3 | Left WL3, 4 | 60 | - | - | - | - | - | - |
|  | IGPB-AR-340 | 1 | body, dorsal | 60 | fig. 5 C, F, I | 11 | 62 | 140 | -0.59 | 0.13 |
|  |  | 2 | left WL1, dorsal view | 60 | fig. 15 L-M | - | - | - | - | - |
|  |  | 3 | Left WL2, 3 | 60 | - | - | - | - | - | - |
|  |  | 4 | Right WL1, dorsal view | 60 | - | - | - | - | - | - |
|  |  | 5 | Right WL2, 3 | 60 | - | - | - | - | - | - |
|  | IGPB-M-142 | - | Cephalon and trunk (part) | 60 | - | - | - | - | - | - |
|  | IGPB-HS206 | 1 | cephalon and trunk, dorsal | 60 | fig. 7 A-C | 95 | 78 | 54 | -0.53 | -0.12 |
|  |  | 2 | left WL1 | 60 | - | - | - | - | - | - |
|  |  | 3 | left WL4 | 60 | - | - | - | - | - | - |
|  |  | 4 | abdomen (dorsal) and right WL2-4 | 60 | - | - | - | - | - | - |
|  |  | 5 | right WL1-4 | 60 | - | - | - | - | - | - |
|  | IGPB-HS207 | 1 | trunk and cephalon, ventral | 60 | fig. 3 A, E, I | 11 | 70 | 31 | 0.27 | 0.28 |
|  |  | 2 | trunk and abdomen, ventral | 60 | - | - | - | - | - | - |
|  |  | 3 | right WL2 | 60 | - | - | - | - | - | - |
|  |  | 4 | right WL1, proximal | 60 | - | - | - | - | - | - |
|  |  | 5 | right WL1, distal | 60 | - | - | - | - | - | - |
|  | IGPB-HS456 | 1 | cephalon and trunk, dorsal | 60 | - | - | - | - | - | - |
|  |  | 2 | abdomen and left WL3-4, dorsal | 60 | - | - | - | - | - | - |
|  | IGPB-HS457 | 1 | body and proboscis (?), lateral | 40 | fig. 4 A, C | - | - | - | - | - |
|  |  | 2 | right WL1-2 | 60 | - | - | - | - | - | - |
|  | IGPB-HS582 | 1 | specimen 1, cephalon, dorsal | 60 | fig. 10 B-C | - | - | - | - | - |
|  |  | 2 | specimen 1, distal podomeres of left WL1 | 60 | fig. 15 I, J | - | - | - | - | - |
|  |  | 3 | specimen 1, right WL2 | 60 | fig. 16 M-O | 3 | 98 | 1 | -0.19 | 0.24 |
|  |  | 4 | specimen 1, WL2-4 and telson | 60 | - | - | - | - | - | - |
|  |  | 5 | specimen 2, right WL2-3 | 60 | fig. 16 E-G | 0 | 71 | 7 | -0.18 | 0.22 |
|  |  | 6 | specimen 2, right WL4 | 60 | - | - | - | - | - | - |
|  | IGPB-HS636 | 1 | right WL1 | 40 | fig. 15 E-G | 15 | 25 | 150 | -0.06 | -0.60 |
|  |  | 2 | cephalon and trunk, dorsal | 60 | - | - | - | - | - | - |
|  |  | 3 | abdomen, dorsal | 60 | - | - | - | - | - | - |
|  |  | 4 | WL2-4 right | 60 | - | - | - | - | - | - |
|  | IGPB-HS660 | 1 | abdomen, ventral | 60 | fig. 8 M-O | 56 | 100 | 1 | 0.31 | 0.34 |
|  |  | 2 | cephalon, ventral | 60 | fig. 3 B, F, J | 5 | 88 | 7 | -0.22 | -0.30 |
|  |  | 3 | body, left WL1-4 | 40 | - | - | - | - | - | - |
|  | IGPB-HS694 | 1 | abdomen, lateral | 60 | fig. 9 J, K | - | - | - | - | - |
|  |  | 2 | trunk and cephalon, dorsolateral | 60 | - | - | - | - | - | - |
|  | IGPB-HS942 | 1 | whole plate (also including Pycnogonida indet.) | 60 | fig. 17 A, C, E | 20 | 50 | 95 | 0.13 | -0.39 |
|  |  | 2 | specimen 1, upper part | 105 | - | - | - | - | - | - |
|  |  | 3 | specimen 1, lower anterior part | 105 | - | - | - | - | - | - |
|  |  | 4 | specimen 1, lower posterior part | 105 | - | - | - | - | - | - |
|  | IGPB-HS1039 | 1 | specimen 1, abdomen, lateral | 60 | fig. 8 I-K | 8 | 91 | 6 | 0.06 | 0.47 |
|  |  | 2 | specimen 1, WLs left 2-4, right 4 | 60 | fig. 16 A-C | 8 | 80 | 2 | -0.27 | -0.16 |
|  |  | 3 | specimen 1, whole specimen | 40 | - | - | - | - | - | - |
|  |  | 4 | specimen 2, cephalon and trunk, dorsolateral | 60 | - | - | - | - | - | - |
|  |  | 5 | specimen 2, right WL1 | 40 | - | - | - | - | - | - |
|  |  | 6 | specimen 3, abdomen, lateral | 60 | - | - | - | - | - | - |
|  | MB-A-46 | 1 | abdomen, dorsal | 60 | fig. 8 A-C | 40 | 80 | 71 | 0.13 | -0.43 |
|  |  | 2 | left WL1 | 60 | fig. 15 O, P | - | - | - | - | - |
|  |  | 3 | right WL4 | 60 | fig. 16 I-K | 0 | 69 | 18 | 0.13 | 0.70 |
|  |  | 4 | trunk and cephalon, dorsal | 60 | - | - | - | - | - | - |
|  | MB-A-47 | 1 | cephalon, trunk and abdomen, dorsal | 60 | - | - | - | - | - | - |
|  |  | 2 | left WL1 | 60 | - | - | - | - | - | - |
|  | MB-A-288 | 1 | trunk, proximal WL | 60 | - | - | - | - | - | - |
|  |  | 2 | distal WL | 60 | - | - | - | - | - | - |
|  | MB-A-313 | - | fragment of the trunk | 60 | - | - | - | - | - | - |
|  | MB-A-3969 | - | cephalon and first trunk segment | 60 | - | - | - | - | - | - |
| *Palaeopantopus maucheri* | SNSB-BSPG 1930 I 501 | 1 | body, lateroposterior | 100 | fig. 21 A-C | 50 | 70 | 105 | 0.19 | 0.54 |
|  |  | 2 | upper part of the fossil | 50 | fig. 23 P-R | 0 | 62 | 126 | -0.83 | 00.6 |
|  |  | 3 | lower part of the fossil | 50 | - | - | - | - | - | - |
|  | SNSB-BSPG 1929 V 3 | 1 | body, dorsal | 60 | fig. 20 A-C | 40 | 70 | 75 | -0.47 | 0.04 |
|  |  | 2 | right WL | 60 | fig. 23 A-C | 25 | 57 | 75 | -0.34 | -0.29 |
|  |  |  |  |  | fig. 23 D-F | 14 | 50 | 20 | 0.08 | 0.06 |
|  |  |  |  |  | fig. 23 G-I | 4 | 52 | 13 | 0.62 | -0.04 |
|  |  |  |  |  | fig. 23 J-L | 4 | 55 | 18 | 0.53 | -0.28 |
|  |  | 3 | body (dorsal) and left WL | 105 | - | - | - | - | - | - |
|  |  | 4 | trunk (ventral), proboscis and cephalic appendages | 105 | fig. 20 E-F | - | - | - | - | - |
|  | MB-A-45 | 1 | body, ventral | 105 | fig. 22 A-C | 31 | 70 | 36 | -0.14 | 0.56 |
|  |  | 2 | left WL3-4 | 105 | fig. 23 M-O | 0 | 72 | 45 | -0.10 | -0.60 |
|  |  | 3 | whole specimen | 60 | - | - | - | - | - | - |
| Pycnogonida gen. sp. | IGPB-HS437 | 1 | whole specimen, ventral | 60 | fig. 32 A-B | - | - | - | - | - |
|  |  | 2 | body, ventral | 105 | fig. 33 A-C | 13 | 54 | 61 | -0.01 | -0.03 |
| *Flagellopantopus blocki* | NHMMZ PWL 2004/5024-LS | 1 | whole specimen (flagellum incomplete), ventral | 60 | fig. 25 A-B | - | - | - | - | - |
|  |  | 2 | proboscis, ovigers, WL1-2 | 105 | fig. 26 A-D | - | - | - | - | - |
|  |  | 3 | left WL3-4 | 105 | - | - | - | - | - | - |
| *Pentapantopus vogteli* | NHMMZ PWL 2010/5-LS | 1 | full specimens 1 and 2, ventral and lateral | 105 | fig. 28 A-B | - | - | - | - | - |
|  |  | 2 | whole fossil | 60 | - | - | - | - | - | - |
| *Pentapantopus*? *vogteli*? | NHMMZ PWL 2007/29-LS | 1 | whole specimen, dorsal | 105 | fig. 30 B-D | 10 | 70 | 77 | 0.26 | 0.42 |
|  |  | 2 | specimen and associated ophiuroids | 60 | - | - | - | - | - | - |
| Pycnogonida indet. | NHMMZ PWL 2010/5-LS | 3 | specimen 3, whole specimen, lateral? | 105 | fig. 34 A-C | 22 | 30 | 90 | -0.12 | -0.22 |
|  |  | 4 | specimen 4 whole specimen, lateral? | 105 | fig. 34 E-G | 21 | 80 | 75 | 0.24 | 0.09 |
|  | IGPB-HS942 | 5 | whole specimen, lateral? | 105 | fig. 34 I-K | 13 | 54 | 78 | 0.29 | -0.23 |
